# Microbiomes in Canidae

**DOI:** 10.1002/ece3.8449

**Published:** 2021-12-16

**Authors:** Tyler L. Biles, Harald Beck, Brian S. Masters

**Affiliations:** ^1^ Department of Biological Sciences Towson University Towson Maryland USA

**Keywords:** anthropogenic stress, canids, coyote, dysbiosis, gut microbiome, red fox

## Abstract

Because of their range expansion across North America, coyotes (*Canis latrans*) now occur sympatrically with numerous other predator species, including red foxes (*Vulpes vulpes*). This raises several interesting ecological questions, including if and how sympatry affects the diet and gut microbiomes of coyotes and red foxes. We examined the gut microbiomes of sympatric populations of coyotes and red foxes within two different National Parks in Virginia, USA, that differ in land use, vegetation, and anthropogenic disturbance: Prince William Forest Park (PRWI) and Manassas National Battlefield Park (MANA). From 2012 to 2017, scat samples from PRWI and MANA were collected and analyzed. Polymerase chain reaction (PCR) amplification of a region of the mitochondrial cytochrome‐b gene followed by restriction enzyme digestion of the PCR product was used to determine the origin of each scat sample. Next‐Generation DNA sequencing of a hypervariable 16S rRNA gene region was used to determine gut microbiome information about the scat samples. There was no evidence for a difference between the gut microbiomes of red foxes in either location, or for a difference between the gut microbiomes of red foxes at either location and coyotes at the location with lower human disturbance, PRWI. However, the gut microbiomes of coyotes at the location with higher anthropogenic disturbances, MANA, revealed a marked change from those found in red foxes at either location and from those in coyotes at the location with lower disturbances. The gut microbiomes of coyotes subjected to greater human impact may provide evidence of dysbiosis, indicative of increased physiological stress and reduced health. We discuss our observations in the context of understanding anthropogenic impacts on coyote and red fox interactions. Our results suggest that physiological stress in the form of human disturbance may play an important role in the composition of the gut microbiome of coyotes, which can affect their overall health.

## Graphical abstract

In this study, we examined the gut microbiomes of sympatric populations of coyotes (*Canis latrans*) and red foxes (*Vulpes vulpes*) from two US National Parks, each with a different level of human disturbance. We used Next‐Generation DNA sequencing of a hypervariable 16S rRNA gene region to determine the composition of the gut microbiome of coyotes and red foxes. Some of our findings indicated that while the gut microbiomes of red foxes did not differ between the two National Parks, it was distinct for coyotes between the two parks. Thus, we provide evidence that human disturbance could lead to dysbiosis, physiological stress, and reduced health in coyotes.

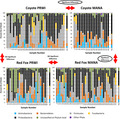

## 1 INTRODUCTION

There has been considerable interest in the ecological impact of the geographic expansion of coyotes (*Canis latrans*) across North America in both natural settings and anthropogenically altered systems. Because coyotes readily prey on domestic cats (*Felis catus*) (Crooks & Soule, 1999; Grubbs & Krausman, 2009), they can reduce the negative impact of feline predation pressure on vertebrate populations (Crooks & Soule, 1999). Considering that cats may kill up to 4 billion birds and 22.3 billion mammals in the United States annually (Loss et al., 2013), coyotes clearly could contribute to the maintenance of vertebrate diversity.

The presence of coyotes also has a predictable impact on red foxes (*Vulpes vulpes*). At the scale of the entire North American continent, interactions involving wolves, coyotes, and foxes result in coyotes being more abundant than foxes in areas without wolves, and foxes being more abundant than coyotes in areas where wolves are present, which are two examples of mesopredator release (Newsome & Ripple, 2015). In Minnesota, the reintroduction of wolves has had the effect of suppressing coyote populations to the benefit of fox populations (Levi & Wilmers, 2012). In areas where coyotes and foxes are sympatric in the absence of wolves, humans seem to be playing a mediating role, much as has been seen in coyote and cat interactions. Multiple studies have found that coyotes avoid human development, while foxes avoid coyotes and tend to concentrate in areas that are highly developed (Cove et al., 2012; Moll et al., 2018; Wait et al., 2018). Observations by Mueller et al. (2018) indicate that coyote/fox interactions are not necessarily antagonistic and harmful to foxes, but their data also show foxes and coyotes partitioning use of urban landscape, with foxes occupying more developed areas, and coyotes avoiding areas heavily used by humans.

Coyotes in poor health (where state of health was assessed by the visible presence of sarcoptic mange) were more likely to use human‐developed areas (Murray et al., 2015), and the authors speculated that diseased coyotes might enter urban habitats in search of more easily obtained anthropogenic food sources. Developments in DNA sequencing technology allow a unique and non‐invasive approach to examining the health of animals in the wild, and the effects of environment and ecology through the analysis of their gut microbiomes (Bahrndorff et al., 2016; Barko et al., 2018; Ingala et al., 2018). Recent research has shown the microbiome to be critical to health in humans (The Integrative Human Microbiome Project Research Network Consortium, 2014), and this understanding has been extended to other species (Swanson et al., 2011). Increasingly, the gut microbiome is being studied to examine its role in health and disease in a range of non‐human animals (Hanning & Diaz‐Sanchez, 2015), providing new opportunities to explore the impact of a wide range of ecological interactions on wildlife. Here we describe our analysis of microbiomes of coyotes and foxes living in sympatry in two different National Parks representing very different levels of human habitation, development, and land use.

## 2 MATERIALS AND METHODS

### 2.1 Study sites

The study sites were Prince William Forest Park (PRWI) and Manassas National Battlefield Park (MANA), both located in Prince William County, Virginia, USA and part of the United States’ National Park Service (NPS) system (Lookingbill et al., 2014). PRWI, an area of relatively low human disturbance, contains about 5075 hectares of undeveloped deciduous forest, with hiking trails and campgrounds present (Bozarth et al., 2011). MANA is about 30 km north of PRWI and comprises around 2100 hectares of open fields and forested areas (Rossell et al., 2005). A site of high human activity and disturbance, MANA contains many walking trails, open‐air historic monuments, and picnic areas for visitors, has a major highway and other roads passing through it, and is surrounded by commercial and residential areas (Rossell et al., 2005).

### 2.2 Scat collection

From 2012 to 2017, scat samples were collected across different seasons from both PRWI and MANA (permit PRWI‐2013‐SCI‐0002). Scat samples were collected either through the use of a grid system, which involved randomly choosing a plot area and then walking trails within that plot area to collect samples, or by walking various trails within the parks. Because aging of samples and potential contamination by microbes is difficult to assess in the field, we disregarded samples with visible microbial or fungi growth, and did not collect immediately after rain. A total of 264 scat samples were gathered. Each scat sample was divided into subsamples for analyses in different labs at Towson University and stored at −20°C.

### 2.3 DNA isolation

We used QIAamp DNA Stool Mini Kits (QIAGEN, Inc., Valencia, California, USA) to isolate total DNA from each scat sample. The Stool Pathogen Detection protocol, along with the recommended steps, was followed. Isolated DNA was stored at −20°C and used in downstream applications.

### 2.4 Identifying canid species

To determine which canid species produced the scat samples, we followed a protocol described by Paxinos et al. (1997) to PCR amplify a ~400 bp region of the mitochondrial cytochrome‐b gene. Subsequent restriction enzyme digestion of the PCR product yielded an agarose gel banding pattern that was species‐specific. To confirm the results of the restriction enzyme digestions, we used a protocol developed by Bozarth et al. (2010). We PCR amplified a portion of the mitochondrial d‐loop region and the resulting PCR products were sized on agarose gels. In this case, PCR product size allowed species identification.

### 2.5 Determining the gut microbiome

To determine the bacteria community present in the gut of the canid species, we used primers to PCR amplify a hypervariable V3 and V4 region of the 16S rRNA gene (Klindworth et al., 2013) in DNA isolated from the scat samples. We followed the protocol described in Illumina's 16S Metagenomic Sequencing Library Preparation guide (https://support.illumina.com/downloads/16s_metagenomic_sequencing_library_preparation.html).

### 2.6 DNA sequencing

Following amplification of a region of the 16S rRNA gene for all samples, we used a unique pair of index primers for each sample in a second PCR step, following the protocol described in Illumina's 16S Metagenomic Sequencing Library Preparation guide. We then pooled samples and sequenced them on an Illumina MiSeq Next‐Generation DNA sequencer using a 2x250 cycle Nextera XT sequencing kit (both from Illumina, Inc., San Diego, California, USA) and following the protocol described in Nextera XT Preparing DNA Libraries for Sequencing on the MiSeq (https://support.illumina.com/downloads/prepare_libraries_for_sequencing_miseq_15039740.html).

Of note, DNA isolation and the first PCR set‐up were both done in a different laboratory from all subsequent post‐PCR activity to avoid potential contamination.

### 2.7 Analyses

We used Illumina's 16S Metagenomics application found on BaseSpace to analyze our sequencing data. This application compares the sample sequences to reference sequences in the Greengenes database to classify the bacteria at different taxonomic levels (https://support.illumina.com/downloads/16s_metagenomic_sequencing_library_preparation.html). For each sample, this application reports the percent of sequence reads corresponding to a specific bacterial taxonomic group. In this study, the identification of the bacteria is reported at the phylum level and family level.

The sequencing data, specifically the percent of sequence reads in a sample that correspond to a specific bacterial phylum, were compared among the same canid species between the two different parks (PRWI and MANA) and between the two different canid species (coyote and red fox) within the same park. Multivariate analyses involving distance measures were used to determine if there was an association between the gut microbiome and a particular canid species and park, with a significant *p*‐value of .05 or less (McCune & Grace, 2002; Suchodolski, 2016). We used the software program PC‐ORD to carry out Multi‐response Permutation Procedures (MRPP), which is a nonparametric procedure that can be used to test whether a significant difference exists between two groups of sampling units (the scat samples in this case) (Shin et al., 2015), allowing for the comparison of gut microbiome between canid species and park. PC‐ORD also calculates the species richness (S), species evenness (E), Shannon's diversity index (H), and Simpson's diversity index (D) for various group (canid and park) comparisons. In addition, we used Kruskal–Wallis rank sum tests to compare the Shannon diversity and microbial species richness between host species and park. Illumina's 16S Metagenomics application provided the Shannon diversity index for each sample, as well as the number of microbial species present in each sample; these values were used in the Kruskal–Wallis rank sum tests.

For the gut microbiome analyses using MRPP, two matrices were created, with the main matrix containing rows for the scat samples and columns for the bacterial phyla, which are categorical variables. Seven bacterial phyla were included, which were Actinobacteria, Bacteroidetes, Firmicutes, Fusobacteria, Proteobacteria, Unclassified at Phylum level, and Other Phyla. The percent of sequence reads corresponding to each phylum per sample was inserted into the main matrix. The secondary matrix contained rows for the scat samples and a column for the park or canid species, depending on the comparison. For the gut microbiome analyses, the Euclidean (Pythagorean), Jaccard, and Sorensen (Bray–Curtis) distance measures were each used. The output of the MRPP included the average distance (a measure of heterogeneity) within each group, the test statistic (T), the observed and expected delta (δ), the chance‐corrected within‐group agreement (A), and the *p*‐value, with a *p*‐value ≤ .05 indicating significance.

## 3 RESULTS

### 3.1 Samples

The DNA isolations were performed on a total of 198 scat samples collected from PRWI and MANA. Of note, 66 of the 264 collected samples did not undergo the DNA isolation procedure; these samples in many cases contained an insufficient amount of digested material for the procedure (e.g., consisted of lots of hair, bone, or other material hard to digest). Of the 198 scat samples subjected to the DNA isolation procedure, a total of 161 samples were successfully PCR‐amplified, sequenced, and subsequently analyzed to determine the gut microbiome of coyotes and red foxes from the two parks. Of note, 37 of these 198 scat samples could not be PCR‐amplified despite repeated attempts, possibly because the DNA isolation procedure with these samples was unsuccessful due to sub‐optimal amounts of scat being used during the procedure or possibly because these samples contained degraded bacterial DNA. Of the 161 scat samples that were analyzed, 58 were produced by coyotes, and 67 were from red foxes. Three scat samples were from gray foxes (*Urocyon cinereoargenteus*), eight samples came from domestic dogs (*Canis lupus familiaris*), one sample was from a domestic cat (*Felis catus*), and one sample was from a bobcat (*Lynx rufus*). The source of 23 of the 161 samples could not be identified.

### 3.2 Coyote gut microbiome results

**FIGURE 1:**
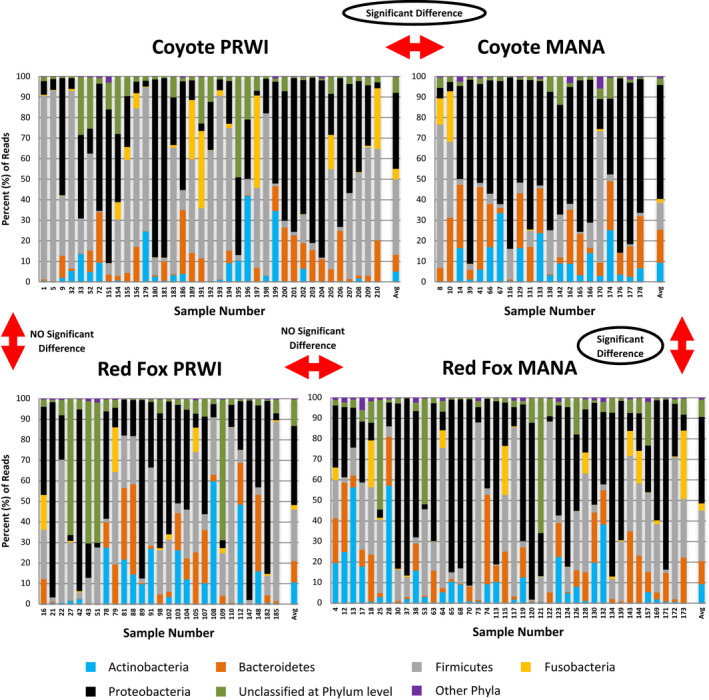
The bacterial phyla present in coyote and red fox scat samples from Prince William Forest Park (PRWI) and Manassas National Battlefield Park (MANA), shown as percent (%) of sequence reads in each sample. The average (Avg) of the percent of reads was calculated across samples for each canid and park. Based on Multi‐response Permutation Procedures (MRPP) performed using PC‐ORD, the composition of the gut microbiome is significantly different between coyotes from both parks and between MANA coyotes and MANA red foxes

Of the 58 coyote gut microbiome samples, 37 were from PRWI and 21 from MANA. Figure 1 shows the percent of reads corresponding to each bacterial phylum in the coyote scat samples from each park. The bacterial phyla Firmicutes or Proteobacteria were the most abundant identified phyla, in terms of percent of sequence reads, in most of the PRWI samples. Among the PRWI coyote samples containing the bacterial phylum Firmicutes, some of the families more commonly identified were Clostridiaceae, Lachnospiraceae, and Planococcaceae, occurring in varying abundance across samples. Among the PRWI coyote samples containing the bacterial phylum Proteobacteria, family Moraxellaceae, Oxalobacteraceae, and Pseudomonadaceae were some of those more commonly identified, though abundance varied across samples. Proteobacteria was also the most abundant identified phylum in most of the MANA coyote samples. Among these samples, some of the bacterial families identified were Moraxellaceae, Oxalobacteraceae, and Sphingomonadaceae in varying abundance across samples. In both the PRWI and MANA samples, the phylum Bacteroidetes was never identified as the most abundant bacterial phylum and Actinobacteria or Fusobacteria were seldom the most abundant phyla in the PRWI samples and were not identified as most abundant in any of the MANA samples.

When averaging the percent of sequence reads across coyote scat samples from each park, the following results were obtained. With the PRWI coyote samples, the average percent reads identified to each major bacterial phylum were: 37.01% for Proteobacteria, 36.64% for Firmicutes, 8.37% for Bacteroidetes, 5.12% for Fusobacteria, and 4.94% for Actinobacteria. The average Shannon diversity was 2.405, while the average number of microbial species in the PRWI coyote scat samples was 198.6. With the MANA coyote samples, the average percent reads were: 55.47% for Proteobacteria, 16.03% for Bacteroidetes, 13.27% for Firmicutes, 9.25% for Actinobacteria, and 1.87% for Fusobacteria. The average Shannon diversity was 2.685, while the average number of microbial species in the MANA coyote scat samples was 302.4.

When comparing the gut microbiome of coyotes from PRWI and MANA, the MRPP statistical results when using each of three distance measures (Euclidean, Jaccard, and Sorensen) showed a *p*‐value << .05, indicating that the gut microbiome of coyotes between both parks was significantly different (Tables 1 and 2).

**TABLE 1:** Summary of statistical results obtained from the canid gut microbiome analyses performed using Multi‐response Permutation Procedures (MRPP) in PC‐ORD

Gut microbiome comparison	Distance measure	Average distance within group		Test statistic (T)	Observed delta (δ)	Expected delta (δ)	Chance‐corrected within‐group agreement (A)	*p*‐value
		Group 1	Group 2					
PRWI Coyote vs. MANA Coyote (58 scats by 7 bacterial phyla)		**PRWI Coyote**	**MANA Coyote**					
	Euclidean	59.494	43.317	−5.444	53.637	57.208	0.062	.002
	Jaccard	0.653	0.523	−5.510	0.606	0.635	0.046	.002
	Sorensen	0.523	0.390	−5.647	0.474	0.506	0.062	.002
PRWI Red Fox vs. MANA Red Fox (67 scats by 7 bacterial phyla)		**PRWI Red Fox**	**MANA Red Fox**					
	Euclidean	66.200	59.287	0.985	62.073	61.564	−0.008	.956
	Jaccard	0.697	0.657	0.956	0.673	0.669	−0.006	.881
	Sorensen	0.570	0.524	0.904	0.542	0.538	−0.007	.871
PRWI Coyote vs. PRWI Red Fox (64 scats by 7 bacterial phyla)		**PRWI Coyote**	**PRWI Red Fox**					
	Euclidean	59.494	66.200	−0.900	62.323	62.830	0.008	.147
	Jaccard	0.653	0.697	−1.117	0.671	0.676	0.007	.123
	Sorensen	0.523	0.570	−0.735	0.543	0.546	0.006	.178
MANA Coyote vs. MANA Red Fox (61 scats by 7 bacterial phyla)		**MANA Coyote**	**MANA Red Fox**					
	Euclidean	43.317	59.287	−2.106	53.789	54.902	0.020	.045
	Jaccard	0.523	0.657	−2.426	0.611	0.622	0.017	.031
	Sorensen	0.390	0.524	−2.449	0.478	0.489	0.023	.031

Note

The distance measures and groups used, as well as the values for the average distance within group, the test statistic (T), the observed delta (δ), the expected delta (δ), and the chance‐corrected within‐group agreement (A), and the *p*‐value are shown for each gut microbiome comparison. The gut microbiome of coyotes between PRWI and MANA, red foxes between PRWI and MANA, coyotes and red foxes in PRWI, and coyotes and red foxes in MANA was analyzed. The distance measures used were Euclidean (Pythagorean), Jaccard, and Sorensen (Bray‐Curtis).

Abbreviations: MANA, Manassas National Battlefield Park; PRWI, Prince William Forest Park.

**TABLE 2:** Additional statistical values from the canid gut microbiome analyses

Host and site	Number of samples	Average Shannon diversity	Average microbial species richness
Coyote PRWI	37	2.405	198.6
Coyote MANA	21	2.685	302.4
Red Fox PRWI	27	2.249	182.7
Red Fox MANA	40	2.441	245.4

Kruskal‐Wallis rank sum test results	*p*‐value
Shannon diversity not significantly different between hosts	.504
Shannon diversity not significantly different between sites	.125
Microbial species richness not significantly different between hosts	.820
Microbial species richness significantly different between sites	.001

Note

The number of samples, average Shannon diversity, and average microbial species richness are shown for each host and site combination. The average Shannon diversity and average microbial species richness were calculated based on the Shannon diversity and microbial species richness per sample, as given by Illumina's 16S Metagenomics application. The average was then calculated across samples for the same host and site. Also shown are the results from the Kruskal–Wallis rank sum tests to determine any significance with Shannon diversity between hosts or sites and microbial species richness between hosts or sites. The *p*‐values from this test are shown.

Abbreviations: MANA, Manassas National Battlefield Park; PRWI, Prince William Forest Park.

### 3.3 Red fox gut microbiome results

Of the 67 red fox gut microbiome samples, 27 were from PRWI and 40 were from MANA. Figure 1 shows the percent of reads corresponding to each bacterial phylum in the red fox scat samples from each park. The bacterial phyla Proteobacteria or Firmicutes were the most abundant identified phyla, in terms of percent of sequence reads, in most of the PRWI samples. Considering the PRWI red fox samples, among those containing the bacterial phylum Proteobacteria, family Enterobacteriaceae and Pseudomonadaceae were identified, while among those containing the bacterial phylum Firmicutes, family Clostridiaceae, Lachnospiraceae, and Planococcaceae were identified. These bacterial families occurred in varying abundance across samples. In addition, either Proteobacteria or Firmicutes were also the most abundant bacterial phyla in most of the MANA samples. Among the MANA red fox samples containing Proteobacteria, family Enterobacteriaceae, Moraxellaceae, and Pseudomonadaceae were some of those more commonly identified, occurring in varying abundance across samples. Among the MANA red fox samples containing Firmicutes, some families more commonly identified were Clostridiaceae and Lachnospiraceae, present in different abundance across samples. Furthermore, Actinobacteria or Bacteroidetes were seldom the most abundant phylum in the red fox PRWI or MANA samples, while Fusobacteria was not identified as the most abundant bacterial phylum among any of the red fox PRWI samples.

When averaging the percent of sequence reads across red fox scat samples from each park, the following results were obtained. With the PRWI red fox samples, the average percent of reads identified to each major bacterial phylum were: 38.32% for Proteobacteria, 25.07% for Firmicutes, 10.55% for Actinobacteria, 10.44% for Bacteroidetes, and 2.25% for Fusobacteria. The average Shannon diversity was 2.249, while the average number of microbial species in the PRWI red fox scat samples was 182.7. With the MANA red fox samples, the average percent of reads were: 42.08% for Proteobacteria, 24.65% for Firmicutes, 11.23% for Bacteroidetes, 9.16% for Actinobacteria, and 3.51% for Fusobacteria. The average Shannon diversity was 2.441, while the average number of microbial species in the MANA red fox scat samples was 245.4.

When comparing the gut microbiome of red foxes from PRWI and MANA, the MRPP statistical results when using each of three distance measures (Euclidean, Jaccard, and Sorensen) showed a *p*‐value > .05, indicating that the gut microbiome of red foxes between both parks was not significantly different (Tables 1 and 2).

### 3.4 Comparison of canid gut microbiomes

Overall, when considering the samples individually, the most abundant identified bacterial phylum in the coyote and red fox scat samples was either Proteobacteria or Firmicutes, regardless of the park, for most samples. In addition, Actinobacteria, Bacteroidetes, and Fusobacteria were rarely the most abundant bacterial phyla, regardless of host species or park. When taking into consideration the average percent of reads across samples from each canid species and park group, the highest average belonged to Proteobacteria regardless of host or park. Fusobacteria had either the lowest or second lowest average percent of reads identified in coyote and red fox scat samples from either park.

When comparing the gut microbiome, specifically the composition of the gut bacterial phyla, of coyotes and red foxes from PRWI, the MRPP statistical results when using each of three distance measures (Euclidean, Jaccard, and Sorensen) showed a *p*‐value > .05, indicating that the gut microbiome between coyotes and red foxes in PRWI was not significantly different. When comparing the gut microbiome, specifically the composition of the gut bacterial phyla, of coyotes and red foxes from MANA, the MRPP statistical results when using each of three distance measures showed a *p*‐value < .05, indicating that the differences observed in gut microbiomes between coyotes and red foxes in MANA were unlikely to be due to chance. In addition, based on Kruskal–Wallis rank sum tests, the average Shannon diversity of the gut microbiome was not demonstrably different between hosts or sites, with *p*‐values > .05. Also, based on these tests, the number of microbial species in the scat samples did not differ significantly between hosts (*p*‐value > .05), but it did differ significantly between sites, with a *p*‐value << .05 (Tables 1 and 2).

## 4 DISCUSSION

Based on our analyses of microbiome composition, there was no support for a difference between the gut microbiomes of red foxes at MANA and PRWI, nor between the gut microbiomes of red foxes at either location and coyotes at PRWI. By contrast, the composition of the gut microbiomes of coyotes at MANA were substantially different from the gut microbiomes of coyotes at PRWI and the red foxes at MANA and PRWI. Specifically, the MANA coyote samples were marked by a substantial increase in Proteobacteria, and a substantial decrease in Firmicutes. Firmicutes are typically found in the gut microbiota of humans, dogs, and other mammals, and a decrease in abundance has been proposed as an indicator of dysbiosis (Barko et al., 2018; Litvak et al., 2017). By contrast, while Proteobacteria are also commonly found in the guts of humans, dogs, and other mammals, an increase in Proteobacteria has been proposed as an indicator of dysbiosis (Blanchet et al., 2009; Hollins & Hodgson, 2019). Thus, both notable changes in the gut microbiome of coyotes at MANA are indicative of physiological stress and poor health conditions.

We believe our observations provide additional context to the concept of interacting conditions of stress, low‐quality diet, poor habitat, and decreased resistance to disease described by Murray et al. (2015) regarding urban coyotes in Alberta, Canada. They tracked the behavior of 19 coyotes, 11 apparently healthy and eight with sarcoptic mange. The healthy coyotes tended to avoid areas impacted by human activity and were less likely to consume anthropogenic foods, whereas diseased coyotes were more likely to frequent developed areas and utilized anthropogenic foods. In addition, diseased coyotes assimilated 60% as much dietary protein as healthy coyotes. Murray et al. (2015) hypothesized that coyotes with disease, poor nutrition, or decreased hunting effectiveness may be forced into less desirable habitat that is heavily impacted and developed by humans. It is difficult, however, to know which factors in a complex, interconnected system are causal, which are resultant, and which are incidental (Gao et al., 2018).

We have observed that coyotes (but not red foxes) in areas more heavily utilized by humans show gut microbiome shifts associated with poor health. Multiple studies indicate that coyotes (but not red foxes) prefer to avoid heavily human‐impacted areas (Cove et al., 2012; Moll et al., 2018; Mueller et al., 2018; Wait et al., 2018). It seems likely that coyotes forced into such areas are physiologically stressed, which can result in the gut microbiome changes found in multiple species (Hollins & Hodgson, 2019), which in turn can induce disease. Indeed, experimental manipulation of laboratory mice indicates that many of the deleterious effects of stress are actually caused by changes in the gut microbiome (Gao et al., 2018). Further, genetically identical mice raised under identical conditions, but with differing gut microbiomes, show differing resistance to infectious disease (Velazquez et al., 2019). It is therefore possible that what we have observed is the result of a concatenation of circumstances: coyotes forced into areas they consider sub‐optimal are stressed, which leads to changes in the gut microbiome, which in turn is causal of a diseased state, including a damaged immune response and poor absorption of nutrients. This model is further supported by our data on red foxes. There is no reason to assume red foxes are stressed by occupying areas heavily utilized by humans; multiple studies found that red foxes seek out highly developed areas when coyotes are present (Cove et al., 2012; Moll et al., 2018; Mueller et al., 2018). In our data, coyotes in the more developed MANA had alterations in their gut microbiome consistent with dysbiosis when compared with coyotes in the less‐developed PRWI, but our data provide no evidence that there is any difference between the gut microbiomes of red foxes in the two locations. This may indicate that the human‐impacted landscape has an effect on coyotes that it does not have on red foxes. Further, this observation makes it unlikely that the change seen in coyotes is caused by the presence of anthropogenic toxins, such as anticoagulant rodenticide that has been reported to affect urban bobcat populations (Riley et al., 2007; Serieys et al., 2018), and that has been suggested as a possible cause of anthropogenic stress in coyotes (Murray et al., 2015).

Increasingly, the gut microbiome is being shown to play a highly significant role in health and disease. Here we compared two competing species of canids with marked differences in toleration of human activity. In so doing, we provided a greater insight into the possible significance of the gut microbiome in the health of wild populations (Kartzinel et al., 2019). Our data suggest a working model that makes only a few assumptions that are supported by the literature. Coyotes forced into areas heavily utilized by humans become stressed, altering their gut microbiomes, resulting in poor health, poor nutrition, and an increased susceptibility to disease. We are not suggesting that this model can explain all the results observed when wild animals forced into urban habitats are studied, but we think that it seems likely that the gut microbiome may play a central role in a positive feedback cycle of stress and decreased resistance to disease that should be considered when studying any perturbed population.

## CONFLICT OF INTEREST

The authors declare no conflict of interest.

## AUTHOR CONTRIBUTIONS

**Tyler L. Biles:** Formal analysis (lead); Investigation (equal); Methodology (equal); Writing – review & editing (equal). **Harald Beck:** Conceptualization (equal); Investigation (lead); Project administration (equal); Writing – original draft (equal); Writing – review & editing (equal). **Brian S. Masters:** Conceptualization (equal); Data curation (lead); Formal analysis (equal); Investigation (equal); Methodology (lead); Project administration (equal); Supervision (lead); Validation (lead); Writing – original draft (lead); Writing – review & editing (equal).

## Data Availability

We provide summary data within the manuscript text. Selected course material can be requested directly from the authors.
